# Comparative Analysis of Acquired Resistance to Bortezomib in Prostate Cancer Cells Using Proteomic and Bioinformatic Tools

**DOI:** 10.1111/jcmm.70254

**Published:** 2025-01-12

**Authors:** Semih Seker, Betul Sahin, Azmi Yerlikaya

**Affiliations:** ^1^ Department of Medical Biology, Faculty of Medicine Kutahya Health Sciences University Kutahya Turkey; ^2^ Acibadem Labmed Clinical Laboratories Istanbul Turkey

**Keywords:** bioinformatics, bortezomib, drug resistance, prostate cancer, proteomics

## Abstract

Chemotherapy is a potent tool against cancer, but drug resistance remains a major obstacle. To combat this, understanding the molecular mechanisms behind resistance in cancer cells and the protein expression changes driving these mechanisms is crucial. Targeting the Ubiquitin‐Proteasome System (UPS) has proven effective in treating multiple myeloma and shows promise for solid tumours. Despite initial success with the proteasome inhibitor bortezomib, acquired resistance soon after treatment poses a significant challenge to its efficacy. In this study, we explored proteins potentially involved in acquired resistance to bortezomib using label‐free nLC–MS/MS proteomic analysis. The investigation revealed 299 proteins with notable differences in expression levels in the bortezomib‐resistant PC3 prostate cancer cell line. Using bioinformatics tools, we illustrated the top 10 gene ontology (GO) processes [e.g., translational initiation (*p* = 5.964E‐10), CRD‐mediated mRNA stabilisation (*p* = 1.636E‐5), and hydrogen ion transmembrane transport (*p* = 6.46E‐5)] and the top 20 KEGG [e.g., metabolic pathways (*p* = 7.601E‐13), biosynthesis of amino acids (*p* = 3.834E‐12), and chemical carcinogenesis—reactive oxygen species (*p* = 1.891E‐4)] and REACTOME [e.g., metabolism (*p* = 4.182E‐21), translation (*p* = 9.484E‐18), and Nonsense‐Mediated Decay (NMD) (*p* = 1.829E‐8)] pathways in the PC3‐resistant cells. We further refined our results by comparing them with globally validated TCGA datasets. We correlated the 299 proteins identified through proteomic analysis with tumour aggressiveness and resistance by comparing them with the TCGA nodal metastasis N0 vs. N1 datasets using the UALCAN portal and identified 37 proteins consistent with our results. We believe that a combination of bortezomib with chemotherapeutics targeting these proteins could be effective in overcoming the resistance developed against bortezomib.

## 1 Introduction

The Ubiquitin‐Proteasome System (UPS) is an intracellular protein degradation mechanism that plays a crucial role in cellular homeostasis. It is a critical protein quality control system responsible for the degradation of misfolded proteins resulting from genetic mutations or translational errors [1, 2]. In addition to misfolded and mutated proteins, key regulatory proteins such as transcription factor c‐Fos, phase‐specific cyclins (M, S, and G1 phases), cyclin‐dependent kinase inhibitors, p53, ornithine decarboxylase (ODC), and various oncoproteins are degraded by the UPS [3, 4, 5]. The 26S proteasome, a component of the UPS, consists of two major subunits known as 20S and 19S [6]. The 26S proteasome has three distinct proteolytic activities: chymotrypsin‐like activity (cleaving peptide bonds after Phe, Trp, Tyr, and Leu residues), trypsin‐like activity (cleaving after lysine and arginine residues), and caspase‐like activity (cleaving after acidic amino acids), associated respectively with the β5, β2, and β1 subunits of the 20S proteasome [7, 8].

Under normal physiological conditions, proteins tagged with ubiquitin are degraded by the 26S proteasome [9], while some damaged or misfolded proteins (e.g., ornithine decarboxylase) can also be degraded via a ubiquitin‐independent pathway [10]. Numerous studies have established that the 26S proteasome is involved in almost all cellular processes, including protein degradation, cell cycle regulation, apoptosis, DNA repair, transcription, protein quality control, and synthesis [8]. The proteasome subunit or activity increases in many cancer types (e.g., colon, prostate, melanoma, kidney, lung, and liver) [11, 12, 13]. These findings make the 26S proteasome a promising therapeutic target in cancer treatment.

Inhibition of the proteasome disrupts intracellular protein homeostasis by causing the accumulation of polyubiquitinated proteins, leading to cellular stress and apoptosis [7]. Bortezomib, the first proteasome inhibitor clinically approved by the FDA for the treatment of multiple myeloma [14] and mantle cell lymphoma [15], is a dipeptidyl boronic acid analog derived from leucine and phenylalanine. Bortezomib reversibly binds to the β5 subunit of the 20S proteasome, and to a lesser extent, the β1 subunit, thereby inhibiting the chymotrypsin‐like activity of the proteasome [16, 17]. Proteasome inhibition by bortezomib has been shown to be effective in over 60 human tumour cell lines and has been reported to have varying effects across different cancer types [18]. Combinations of bortezomib with conventional anti‐cancer drugs (such as 5‐fluorouracil, cisplatin, paclitaxel, and doxorubicin) have demonstrated increased anti‐cancer efficacy compared to bortezomib alone [17, 19, 20].

Despite the promising effects of bortezomib in anti‐cancer therapy, drug resistance has been reported. The main mechanisms of bortezomib resistance include mutations in the proteasomal subunit PSMB5, changes in genes and proteins responsible for stress response, cell survival, anti‐apoptotic pathways, and ultimately the development of multidrug resistance [7, 21, 22, 23, 24, 25, 26, 27]. The development of resistance to bortezomib appears to be a significant barrier to its therapeutic use. Understanding the molecular mechanisms of bortezomib resistance in depth is crucial for identifying new target molecules to overcome this resistance and developing combination therapies using chemotherapeutics that target these molecules.

In this study, we aimed to identify differentially expressed proteins involved in resistance to bortezomib in the PC3 prostate cancer cell line and determine their roles in biological processes and therapeutic target potentials. To achieve this goal, we first increased the resistance level of a previously generated bortezomib‐resistant cell line [26] by continuing stepwise exposure of PC3 cells to bortezomib and then identified differentially expressed proteins between parental and resistant cell lines using label‐free nLC‐MS/MS proteomic analysis. Bioinformatic tools were used to investigate the biological processes, KEGG pathways, protein–protein interactions, and their effects on tumour aggressiveness and metastasis involving the differentially expressed proteins. Our proteomic analysis results were compared with globally validated TCGA datasets for further validation.

## 2 Material and Methods

### 2.1 Material

DMEM medium, 10× trypsin solution, penicillin–streptomycin, glucose, fetal bovine serum (FBS), and all other chemicals were purchased from Sigma‐Aldrich. Stericup Filter Units were obtained from Millipore, bovine serum albumin (BSA) was obtained from Bio‐Rad, and WST‐1 (Cat no: 5015944001) was obtained from Roche Pharmaceuticals. Bortezomib was provided by Prof. Dr. Engin Ulukaya (Istinye University, Istanbul, Turkey).

### 2.2 Cell Culture

The PC3 prostate cancer cell line (ATCC Cat# CRL‐1435, RRID:CVCL_0035) was cultured in DMEM medium containing 4.5 g/L glucose, 0.375% sodium bicarbonate, 10% FBS, 100 μg/mL streptomycin, and 100 U/mL penicillin. The culturing process was carried out at 37°C in a humidified atmosphere containing 95% air and 5% CO_2_. To improve the resistance level of bortezomib‐resistant PC3 cell line, cells were gradually re‐exposed to different concentrations of bortezomib ranging from 2.5 nM to 10 nM over a period of 1 month. These resistant PC3 cells were previously exposed to bortezomib ranging from 1 nM to 80 nM over a period of 6 months as detailed in our previous manuscript [26]. Parental cells were passaged and cultured in parallel for experimental optimisation. The parental PC3 cell line was termed PC3‐P, and the bortezomib‐resistant PC3 cell line was termed PC3‐R.

### 2.3 WST‐1 Assay

To determine the IC_50_ values in PC3‐P and PC3‐R cells, 10,000 cells were initially seeded into each well of a 96‐well plate. Subsequently, when the cells in each well reached approximately 70% confluency, they were treated with different concentrations of bortezomib (1 nM, 10 nM, 100 nM, 500 nM, 1 μM, 10 μM, and 100 μM) for 48 h. After this period, the cells were treated with DMEM medium containing 0.5% FBS and 10 mg/mL WST‐1 for 2 h. Following the WST‐1 treatment, the absorbance of each well was read at 450 nm using a microplate reader. The data were analysed using GraphPad Prism 9 software. The IC_50_ values of bortezomib were obtained using log(inhibitor) versus response‐variable slope nonlinear regression [26].

### 2.4 Label‐Free nLC–MS/MS

To identify differentially expressed proteins in PC3‐P and PC3‐R cells, label‐free nLC‐MS/MS analysis was conducted following protein isolation from the resistant and parental cells. In brief, PC3‐P and PC3‐R cells were seeded into 35 × 10 mm petri dishes (100,000 cells/dish) and incubated at 37°C in a humidified atmosphere containing 95% air and 5% CO_2_ until reaching 70% confluency without any drug treatment. Upon reaching this density, the cells were lysed using RIPA buffer containing 0.05 M DTT. Subsequently, protein quantification was performed using the Bio‐Rad Protein Assay method [28]. Tryptic digestion was carried out by adding trypsin (Promega) to the samples at a ratio of 1:100, followed by incubation at 37°C for 18 h. Peptide concentrations were measured using the Quantitative Fluorometric Peptide Assay (Pierce). Prior to label‐free nLC‐MS/MS analysis, peptide concentration was adjusted to 200 ng/μl in 0.1% formic acid. A mixture of 1 μg of tryptic peptides was analysed by nano‐LC‐MS/MS system (Acquity UPLC M‐Class and SYNAPT G2‐Si HDMS; Waters, Milford, MA, USA). Peptide mixtures were loaded onto a trap column (Symmetry C18, 5 μm, 180 μm i.d. 20 mm) and then separated by an analytical column (CSH C18, 1.7 μm, 75 μm i.d. 250 mm) with a 90‐min linear gradient (4%–40% Acetonitrile 0.1% (v/v) FA, 0.300 mL/min flow rate). Glu‐1‐fibrinopeptide‐B at a concentration of 100 fmol/μl was used as a lock mass reference with a flow rate of 0.500 mL/min at 60‐s intervals. SONAR acquisition mode was used for MS data collection with four‐pole ion transmission with a 24 Da wide range. Positive ionisation mode was used for full scan mode in the range of 50–2000 m/z. Progenesis QI (v.4.0, Waters) was used for peptide feature detection, protein identification, and quantification. Processing parameters were set at 60 counts for low‐energy threshold and 10 counts for high‐energy threshold. Samples were normalised by total ion intensity. Expression changes and *p*‐values were calculated using the statistical package available in Progenesis QI for proteomics, and relative quantification was performed using non‐conflicting peptides [29, 30, 31, 32].

### 2.5 Proteomic Data Analysis

The raw data from label‐free nLC‐MS/MS analysis were filtered using GraphPad Prism 9.0 by setting a threshold of *p*‐value < 0.05 (*t*‐test; independent, 2‐tailed, unequal variance) and fold change ≥ 1.5, to identify differentially expressed proteins between PC3‐P and PC3‐R cells. DAVID online tool was employed to determine the cellular components, molecular functions, and biological processes of the differentially expressed proteins [33]. The graphs and heatmaps of the data obtained from DAVID were generated using SRplot [34]. Metascape online tool (www.metascape.com) was utilised to perform Protein–Protein Interaction (PPI) enrichment analysis based on default parameters, followed by the reconstruction of a module network based on selected genomic databases. The resulting network was generated in the Metascape online tool as a subset of proteins forming physical interactions with at least one member of another list. Through the Molecular Complex Detection (MCODE) algorithm, we first identified connected network components, then applied independent pathway and process enrichment analysis to each MCODE component, and limited the functional annotation of the obtained modules to the top three scoring terms based on the *p*‐value. The determination of whether the selected proteins were involved in gene ontologies and participated in a common network was achieved using the Metascape (www.metascape.org) website [35]. The UALCAN website (http://ualcan.path.uab.edu/index.html) was used to compare the expression data with the nodal metastasis statuses (normal‐N0‐N1) of the available genes in The Cancer Genome Atlas (TCGA) project. Then, proteins consistent with nodal metastasis status were compared with UALCAN TCGA correlation datasets [36].

## 3 Results

### 3.1 Identification of IC_50_ Values After Bortezomib Treatment

**FIGURE 1:**
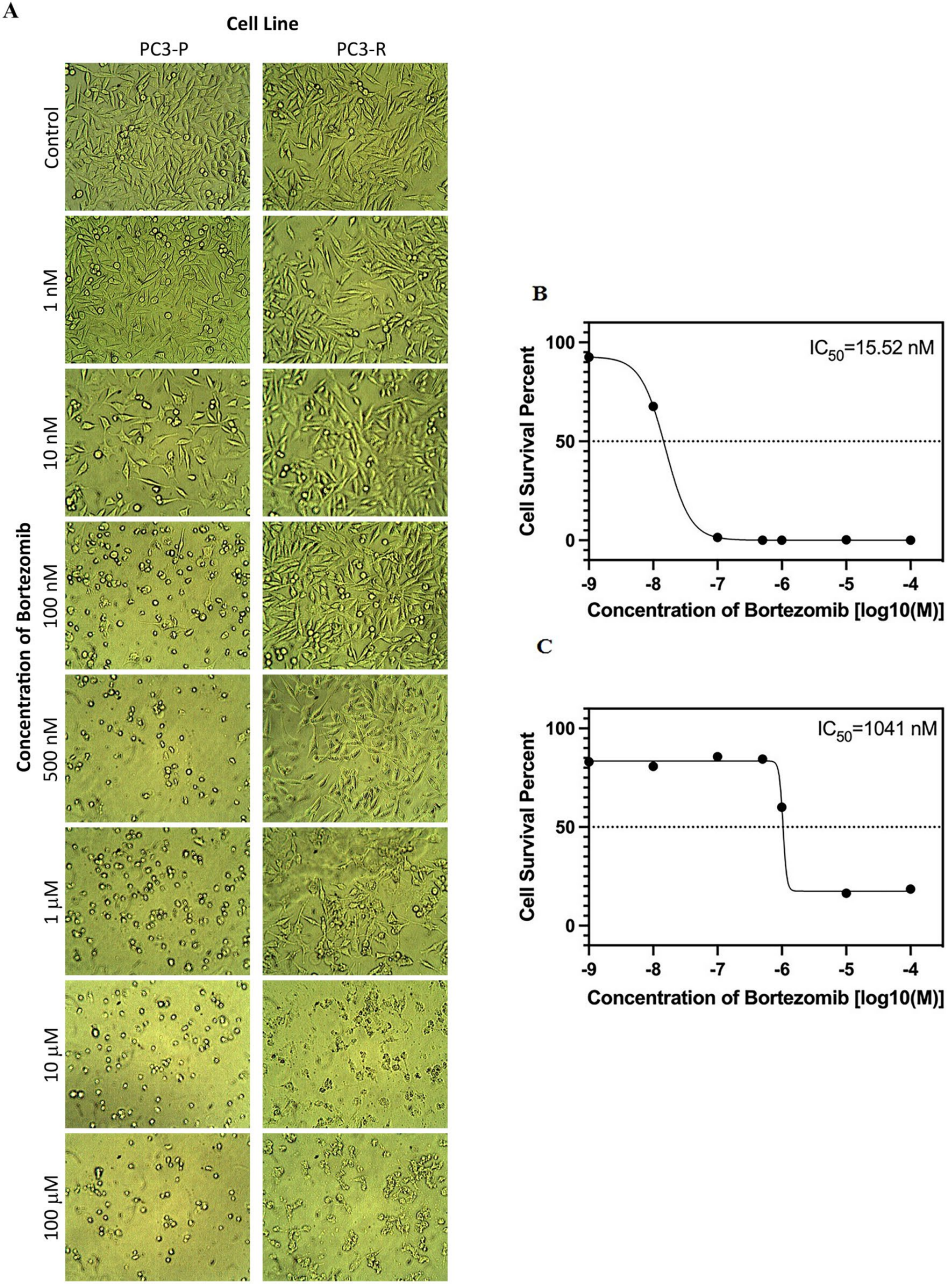
(A) Microscope images of PC3‐P and PC3‐R cells after 48 h of different bortezomib concentrations (viewed under inverted light microscope with 40× magnification). IC_50_ values obtained after treatment of PC3‐P (B) and PC3‐R (C) cells with 1 nM, 10 nM, 100 nM, 500 nM, 1 μM, 10 μM, and 100 μM bortezomib concentrations for 48 h and WST‐1 incubation for 2 h.

One of the biggest problems in cancer treatments is drug resistance that emerges shorty after chemotherapy. Inducing drug resistance through dose escalation in a cell line is a common method used in *in vitro* studies [21, 22, 23, 24, 27, 37]. We previously successfully obtained a bortezomib‐resistant cell line through dose escalation for over 6 months in the PC3 prostate cancer cell line [26]. In this study, we enhanced the resistance level of these cells further by subjecting them to continued treatment with bortezomib concentrations ranging from 2.5 nM to 10 nM for over a month. When examining under the inverted microscope (Figure 1A), it can be observed that after 48 h of bortezomib treatment, the cell density was clearly decreased after treatment with 10 nM bortezomib in PC3‐P cells, while in PC3‐R cells, cells were observed to decreased in number after 1 μM bortezomib concentration treatment, indicating the development of a highly resistance phenotype. Then, using the WST‐1 assay, the IC_50_ value was calculated to quantitavely determine the sensitivity levels of PC3‐P and PC3‐R cells to bortezomib. After a 2‐h WST‐1 incubation, the plate was read at a wavelength of 450 nm, and the data was analysed and found that PC3‐R cells were approximately 67 times more resistant to bortezomib compared to PC3‐P cells (Figure 1B,C).

### 3.2 Identification of Differentially Expressed Proteins

**FIGURE 2:**
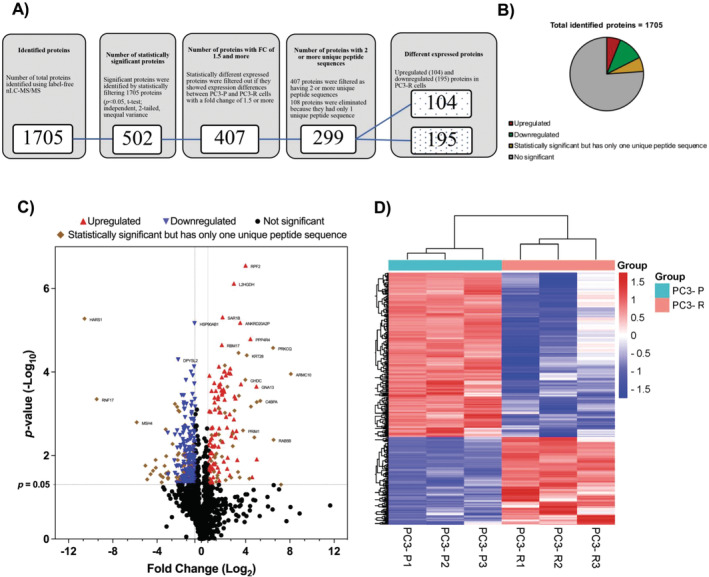
(A) Schematic representation of the step‐by‐step filtering of proteins obtained after label‐free nLC‐MS/MS analysis. (B) Pie chart of total identified and quantified proteins. The expression of 299 (17.5%) from 1705 proteins identified and quantified varied significantly between PC3‐P and PC‐R cell lines. In the PC3‐R cell line, proteins with increased expression are shown in red, proteins with decreased expression are shown in green, proteins that are statistically significant but have 1 unique peptide sequence are shown in orange, and cells without statistically significant expression differences are shown in grey. (C) Volcano plot representation of identified proteins. The x‐axis shows the fold‐changes in protein expression between PC3‐P and PC3‐R, and the y‐axis shows the statistical significance of the differences. The proteins with increased and decreased expression were filtered as *p* < 0.05 and fold change ≥ 1.5 and are shown as red and blue triangles, respectively. Proteins with no significant expression differences between samples are shown as black dots, and proteins with statistically significant expression differences but only one unique peptide sequence are shown as a brown square (drawn with GraphPad Prism 9.0). (D) Heatmap representation of 299 differentially expressed proteins. Blue colour indicates low expression and red colour indicates high expression. Heatmap and cluster analysis was performed with SRplot default settings.

After confirming the resistance levels of the cells, PC3‐P and PC3‐R cells were cultivated without exposure to bortezomib. Upon reaching approximately 70% confluence, the cells were lysed in RIPA buffer supplemented with 0.05 M DTT. Protein quantification was then conducted using the Bradford method in preparation for proteomic analysis. Subsequently, equal amount of protein isolates were subjected to tryptic digestion, followed by label‐free nLC‐MS/MS analysis. The analysis results showed that a total of 1705 proteins were identified from the peptide sequences detected by label‐free nLC‐MS/MS (Table S1). Initially, these 1705 proteins were filtered by selecting the statistically significant ones with a *p*‐value < 0.05 and fold change ≥ 1.5 as well as two or more unique peptide sequences (Figure 2A) to identify proteins differentially expressed in resistant cells. After these filtrations, 299 proteins were identified as differentially expressed between PC3‐P and PC3‐R cells, with 104 proteins showing increased expression in resistant cells and 195 proteins showing decreased expression. Table 1A displays the expression level of significantly upregulated proteins in PC3‐R cells compared to PC3‐P cells, while Table 1B represents the expression level of significantly downregulated proteins. The differentially expressed proteins between PC3‐P and PC3‐R cells were also represented by a pie chart (Figure 2B) and a volcano plot (Figure 2C). In the volcano plot in Figure 2C, the proteins with increased expression in PC3‐R cells are shown as red triangles, the proteins with decreased expression are shown as blue triangles, statistically significant but with only one unique peptide sequence are shown as brown squares (*p* < 0.05; fold change ≥ 1.5). And the proteins with no statistically significant expression differences are shown as black dots. We also visualised the set of 299 differentially expressed proteins between samples with a heatmap (Figure 2D). Upon examining the heatmap, it is evident that the colour scales among sample repetitions are consistent, demonstrating similar expression patterns. Furthermore, distinct expression differences between groups are clearly delineated, and hierarchical clusters in the dendrogram indicate consistent patterns across all samples.

**TABLE 1:** Expression of significantly changed proteins in the resistant cell line as compared to the parental cell line.

Uniprot accession no	Gene ID	Description	Unique peptides	−log10 (*p*)	Fold change (log2) (R/P)*	Molecular weight (Da)
**(A)**						
Q9H7B2	RPF2	Ribosome production factor 2 homologue	5	6.553	3.994	35754.026
Q9H9P8	L2HGDH	L‐2‐hydroxyglutarate dehydrogenase_ mitochondrial	6	6.120	2.947	51057.495
Q9Y6B6	SAR1B	GTP‐binding protein SAR1b	2	5.316	1.919	22523.993
Q5SQ80	ANKRD20A2P	Putative ankyrin repeat domain‐containing protein 20A2	8	5.180	3.519	94999.191
Q6NUP7	PPP4R4	Serine/threonine‐protein phosphatase 4 regulatory subunit 4	2	4.793	4.434	100535.670
Q96I25	RBM17	Splicing factor 45	4	4.650	1.851	45189.707
Q5JPF3	ANKRD36C	Ankyrin repeat domain‐containing protein 36C	6	4.133	1.590	201516.192
Q9Y6Q1	CAPN6	Calpain‐6	4	4.089	2.613	75431.904
P55263	ADK	Adenosine kinase	8	4.017	2.115	40944.691
P84077	ARF1	ADP‐ribosylation factor 1	9	4.015	2.059	20753.842
Q96EY7	PTCD3	Pentatricopeptide repeat domain‐containing protein 3_ mitochondrial	13	3.983	2.472	79234.165
Q14247	CTTN	Src substrate cortactin	4	3.968	2.634	61757.287
Q08211	DHX9	ATP‐dependent RNA helicase A	25	3.925	2.215	142270.353
P00492	HPRT1	Hypoxanthine‐guanine phosphoribosyltransferase	7	3.914	0.733	24807.545
O60841	EIF5B	Eukaryotic translation initiation factor 5B	3	3.878	1.858	139283.262
P31942	HNRNPH3	Heterogeneous nuclear ribonucleoprotein H3	4	3.741	1.173	36983.640
P35241	RDX	Radixin	7	3.716	1.725	68678.072
O94925	GLS	Glutaminase kidney isoform_ mitochondrial	9	3.711	3.581	74316.672
Q8TE68	EPS8L1	Epidermal growth factor receptor kinase substrate 8‐like protein 1	2	3.687	1.603	80536.296
Q14344	GNA13	Guanine nucleotide‐binding protein subunit alpha‐13	2	3.656	4.982	44391.819
P26641	EEF1G	Elongation factor 1‐gamma	16	3.648	1.646	50461.030
P13987	CD59	CD59 glycoprotein	4	3.588	2.007	14804.603
Q9BWJ5	SF3B5	Splicing factor 3B subunit 5	2	3.564	1.818	10249.446
P55010	EIF5	Eukaryotic translation initiation factor 5	8	3.551	1.318	49679.027
P07355	ANXA2	Annexin A2	28	3.551	0.926	38832.220
P11217	PYGM	Glycogen phosphorylase_ muscle form	3	3.535	2.070	97548.453
O60518	RANBP6	Ran‐binding protein 6	3	3.506	1.888	126253.554
P24666	ACP1	Low molecular weight phosphotyrosine protein phosphatase	5	3.464	1.874	18498.735
Q8WXE9	STON2	Stonin‐2	4	3.420	2.996	101906.618
Q92597	NDRG1	Protein NDRG1	10	3.413	1.522	43291.797
P36542	ATP5F1C	ATP synthase subunit gamma_ mitochondrial	9	3.392	1.199	33053.148
P08237	PFKM	ATP‐dependent 6‐phosphofructokinase_ muscle type	7	3.370	3.086	86038.110
P27816	MAP4	Microtubule‐associated protein 4	16	3.356	1.511	121518.447
Q6IBS0	TWF2	Twinfilin‐2	8	3.340	2.104	39776.204
Q16891	IMMT	MICOS complex subunit MIC60	16	3.276	1.196	84077.209
Q5TZA2	CROCC	Rootletin	19	3.156	1.561	228922.725
P12268	IMPDH2	Inosine‐5′‐monophosphate dehydrogenase 2	19	3.107	1.227	56261.312
P30044	PRDX5	Peroxiredoxin‐5_ mitochondrial	10	3.068	0.720	22314.569
Q9H9B4	SFXN1	Sideroflexin‐1	8	3.053	0.841	35904.648
P60660	MYL6	Myosin light polypeptide 6	5	3.040	1.622	17101.181
Q99541	PLIN2	Perilipin‐2	4	3.035	1.728	48303.624
P26196	DDX6	Probable ATP‐dependent RNA helicase DDX6	5	2.994	2.565	54816.116
Q96FQ6	S100A16	Protein S100‐A16	2	2.929	2.236	11858.447
P35580	MYH10	Myosin‐10	9	2.869	2.946	229968.923
Q9ULM3	YEATS2	YEATS domain‐containing protein 2	2	2.679	1.019	151694.317
O95926	SYF2	Pre‐mRNA‐splicing factor SYF2	2	2.674	2.326	28779.448
O95292	VAPB	Vesicle‐associated membrane protein‐associated protein B/C	5	2.659	0.905	27456.560
Q00688	FKBP3	Peptidyl‐prolyl cis‐trans isomerase FKBP3	4	2.655	1.606	25233.885
Q9P2J5	LARS1	Leucine—tRNA ligase_ cytoplasmic	35	2.591	3.170	135664.007
Q14457	BECN1	Beclin‐1	2	2.577	2.828	52409.628
**(B)**						
P08238	HSP90AB1	Heat shock protein HSP 90‐beta	18	5.166	−0.612	83606.509
Q16555	DPYSL2	Dihydropyrimidinase‐related protein 2	6	4.297	−2.096	62749.944
Q9Y2W1	THRAP3	Thyroid hormone receptor‐associated protein 3	2	4.128	−0.654	108722.910
P49448	GLUD2	Glutamate dehydrogenase 2_ mitochondrial	2	4.004	−0.925	61776.265
P62750	RPL23A	60S ribosomal protein L23a	7	3.915	−0.777	17695.083
O00217	NDUFS8	NADH dehydrogenase [ubiquinone] iron–sulfur protein 8_ mitochondrial	4	3.805	−1.393	24218.388
P12004	PCNA	Proliferating cell nuclear antigen	10	3.720	−0.634	29111.015
P33121	ACSL1	Long‐chain‐fatty‐acid—CoA ligase 1	6	3.469	−0.793	78969.986
P14324	FDPS	Farnesyl pyrophosphate synthase	3	3.444	−1.771	48788.796
Q7Z5P4	HSD17B13	beta‐hydroxysteroid dehydrogenase 13	4	3.441	−1.592	33997.657
Q9P289	STK26	STK26_HUMAN Serine/threonine‐protein kinase 26	3	3.349	−0.588	46814.028
P42766	RPL35	60S ribosomal protein L35	4	3.280	−0.858	14551.497
P30837	ALDH1B1	Aldehyde dehydrogenase X_ mitochondrial	11	3.269	−1.578	57662.608
Q99613	EIF3C	Eukaryotic translation initiation factor 3 subunit C	9	3.235	−1.351	106028.340
Q8WUY1	THEM6	Protein THEM6	4	3.222	−1.588	24150.130
Q92973	TNPO1	Transportin‐1	11	3.192	−0.904	103837.953
Q9H361	PABPC3	Polyadenylate‐binding protein 3	2	3.120	−1.500	70259.202
Q04637	EIF4G1	Eukaryotic translation initiation factor 4 gamma 1	13	3.111	−1.551	176232.645
P18669	PGAM1	Phosphoglycerate mutase 1	11	3.037	−0.860	28918.036
P08243	ASNS	Asparagine synthetase [glutamine‐hydrolysing]	4	2.998	−0.880	64940.313
P0DMV8	HSPA1A	Heat shock 70 kDa protein 1A	18	2.916	−1.471	70337.455
P46778	RPL21	60S ribosomal protein L21	4	2.877	−0.694	18621.909
P49006	MARCKSL1	MARCKS‐related protein	5	2.872	−0.931	19585.837
O43175	PHGDH	D‐3‐phosphoglycerate dehydrogenase	20	2.867	−0.708	57391.960
P60891	PRPS1	Ribose‐phosphate pyrophosphokinase 1	5	2.820	−0.679	35347.547
P49756	RBM25	RNA‐binding protein 25	5	2.723	−0.781	100527.802
P17174	GOT1	Aspartate aminotransferase_ cytoplasmic	6	2.698	−1.493	46475.670
O95864	FADS2	Acyl‐CoA 6‐desaturase	2	2.667	−1.618	52373.510
Q9NXF1	TEX10	Testis‐expressed protein 10	2	2.655	−1.207	106415.622
Q9Y2R9	MRPS7	28S ribosomal protein S7_ mitochondrial	2	2.599	−1.564	28248.164
Q9UHB9	SRP68	Signal recognition particle subunit SRP68	3	2.596	−2.185	71242.961
Q02790	FKBP4	Peptidyl‐prolyl cis‐trans isomerase FKBP4	17	2.586	−0.668	52089.786
O95573	ACSL3	Long‐chain‐fatty‐acid—CoA ligase 3	6	2.567	−1.006	81389.711
P02786	TFRC	Transferrin receptor protein 1	25	2.551	−1.010	85327.715
P26358	DNMT1	DNA (cytosine‐5)‐methyltransferase 1	10	2.541	−3.033	185503.613
P52565	ARHGDIA	Rho GDP‐dissociation inhibitor 1	6	2.541	−1.641	23264.168
Q14151	SAFB2	Scaffold attachment factor B2	2	2.540	−0.645	107986.836
Q8IYD1	GSPT2	Eukaryotic peptide chain release factor GTP‐binding subunit ERF3B	2	2.503	−1.081	69510.636
P15090	FABP4	Fatty acid‐binding protein_ adipocyte	4	2.490	−1.911	14832.978
P51570	GALK1	Galactokinase	2	2.469	−0.872	42728.524
Q7Z2Z1	TICRR	Treslin	7	2.430	−0.928	212853.111
P35606	COPB2	Coatomer subunit beta’	7	2.413	−0.643	103342.783
P20700	LMNB1	Lamin‐B1	16	2.395	−0.965	66693.587
Q14694	USP10	Ubiquitin carboxyl‐terminal hydrolase 10	3	2.391	−1.666	87761.183
P62280	RPS11	40S ribosomal protein S11	9	2.368	−1.135	18601.842
P55265	ADAR	Double‐stranded RNA‐specific adenosine deaminase	10	2.337	−1.599	137264.160
P00403	MT‐CO2	Cytochrome c oxidase subunit 2	5	2.337	−1.311	25736.172
P18124	RPL7	60S ribosomal protein L7	12	2.334	−0.647	29282.871
P17858	PFKL	ATP‐dependent 6‐phosphofructokinase_ liver type	2	2.297	−0.622	85817.005
Q9UHD1	CHORDC1	Cysteine and histidine‐rich domain‐containing protein 1	6	2.294	−0.650	38288.197
Q14318	FKBP8	Peptidyl‐prolyl cis‐trans isomerase	4	1.497	−0.691	45018.021

*Note:* (A) Significantly upregulated proteins, (B) Significantly down‐regulated proteins.

* P, PC3‐P cell line; R, PC3‐R cell line.

### 3.3 GO Processes Analysis

**FIGURE 3:**
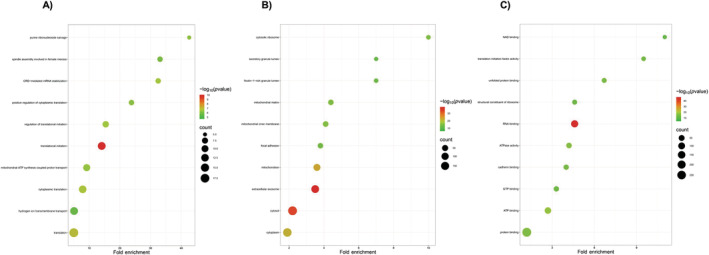
Top 10 GO processes identified by DAVID bioinformatics analysis of different expressed proteins. (A) Biological processes, (B) Cellular Compartments, and (C) Molecular functions (drawn with SRplot).

DAVID online bioinformatics tool was used to determine the biological processes, cellular components, and molecular functions containing differentially expressed proteins based on the *p*‐value, and the top 10 GO processes were graphically represented using SRplot (Figure 3A). In Figure 3A, it is striking that translational processes are the most dramatically affected processes between PC3‐P and PC3‐R cells. Specifically, among the top 10 biological processes with the most significant changes based on *p*‐values between these cell lines, 5 (translational initiation, cytoplasmic translation, translation, positive regulation of cytoplasmic translation, and regulation of translational initiation) of them are found to be related to translational processes. The process of translational initiation (*p* = 5.9637E‐10) is notably affected by acquired resistance to bortezomib. Among the 12 identified proteins (downregulated proteins: EIF3D, EIF4G1, EIF3C, RPS17, EIF4B, EIF3M, EIF4A2, RPS3A, ABCF1, EIF4A1; upregulated proteins: EIF5B, ABCE1) involved in this process, the expression of 10 proteins is decreased in PC3‐R cells, indicating reduced protein synthesis in these cells. In Figure 3B, we depicted the cellular compartments where differentially expressed proteins are found. Interestingly, out of 299 differentially expressed proteins, we identified 115 in extracellular exosomes (*p* = 4.986E‐35). We also determined that 183 differentially expressed proteins are located in the cytosol (*p* = 1.564E‐33), and 80 are found in the mitochondria (*p* = 6.057E‐25). Figure 3C illustrates the top 10 molecular functions in which differentially expressed proteins are involved. The significant changes in various binding functions between the two cells, particularly RNA binding (*p* = 4.243E‐47) molecular function, stand out prominently.

### 3.4 KEGG And REACTOME Pathway Analysis

**FIGURE 4:**
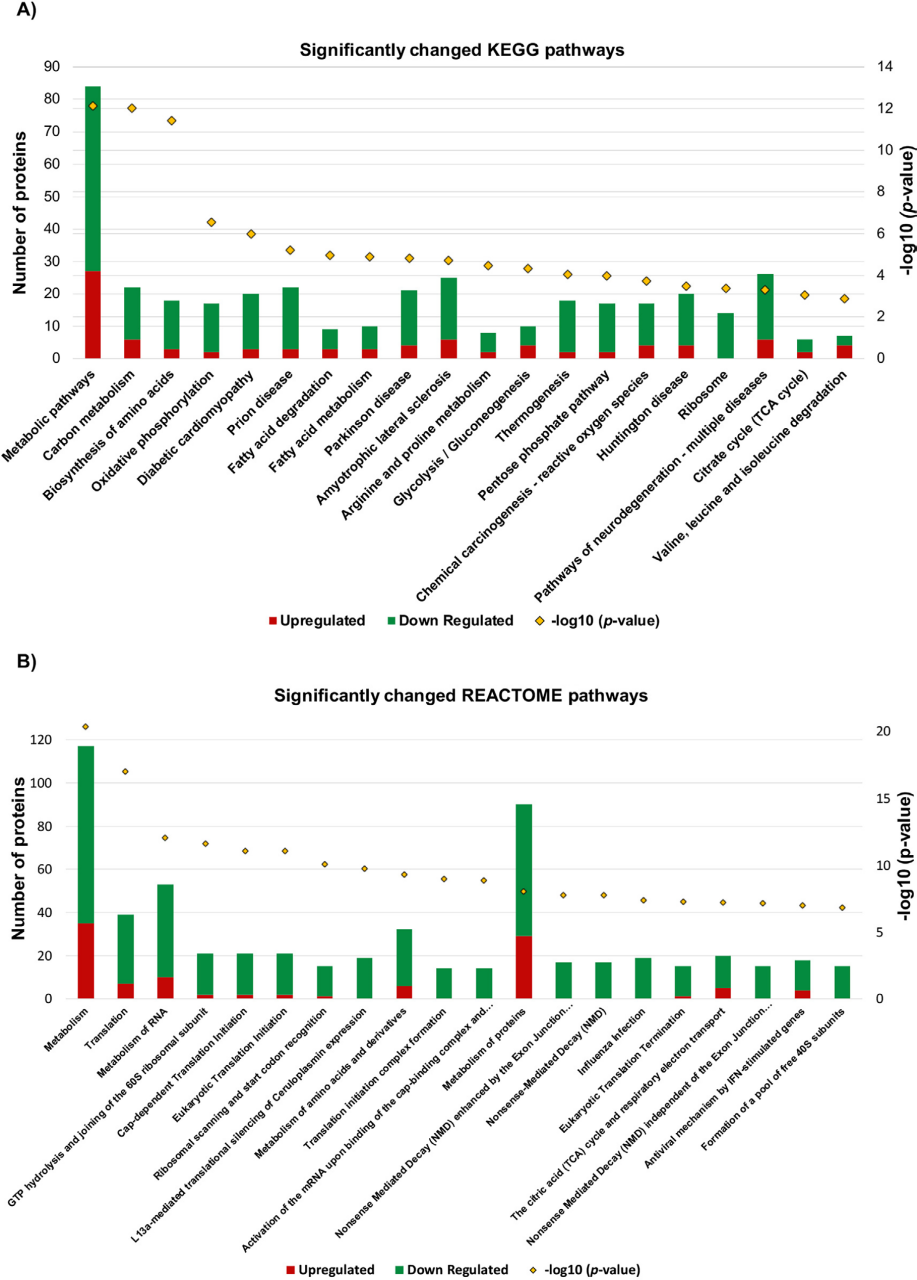
Top 20 KEGG (A) and REACTOME (B) pathways altered between PC3‐P and PC3‐R cells. In both graphs, red colour represents proteins with increased expression, and green colour represents proteins with decreased expression. The *p*‐value of each pathway is shown with a yellow square on the bar.

KEGG (Kyoto Encyclopedia of Genes and Genomes) is a database source that combines genomic, chemical, and systemic functional information [38]. Another database source, the Reactome Knowledge Base, provides a resource that systematically connects human proteins to molecular functions [39]. Using the DAVID bioinformatics tool, we determined which KEGG and Reactome pathway maps the differentially expressed proteins participated in and graphically represented the top 20 KEGG (Figure 4A) and Reactome (Figure 4B) pathways involving differentially expressed proteins in a bar graph. When comparing KEGG pathway and REACTOME pathway analyses, noticeable differences emerge in the types of pathways affected. KEGG pathway analysis highlights changes predominantly in metabolic pathways (e.g., metabolic pathways with *p* = 7.601E‐13 and carbon metabolism with *p* = 9.376E‐13). In contrast, REACTOME pathway analysis reveals alterations not only in metabolism‐related pathways but also prominently in translational processes (e.g., translation with *p* = 9.484E‐18 and Cap‐dependent translation initiation with *p* = 8.300E‐12). The changes identified in translational pathways in the REACTOME analysis are consistent with our GO biological process analyses. Moreover, the discrepancies in the pathways between the two analyses are noteworthy, suggesting that using multiple analysis methods in proteomic studies is essential for obtaining more accurate results. This approach underscores the importance of employing diverse pathway analysis tools to comprehensively capture biological changes and pathways affected in experimental contexts.

### 3.5 Construction of PPI Networks

**FIGURE 5:**
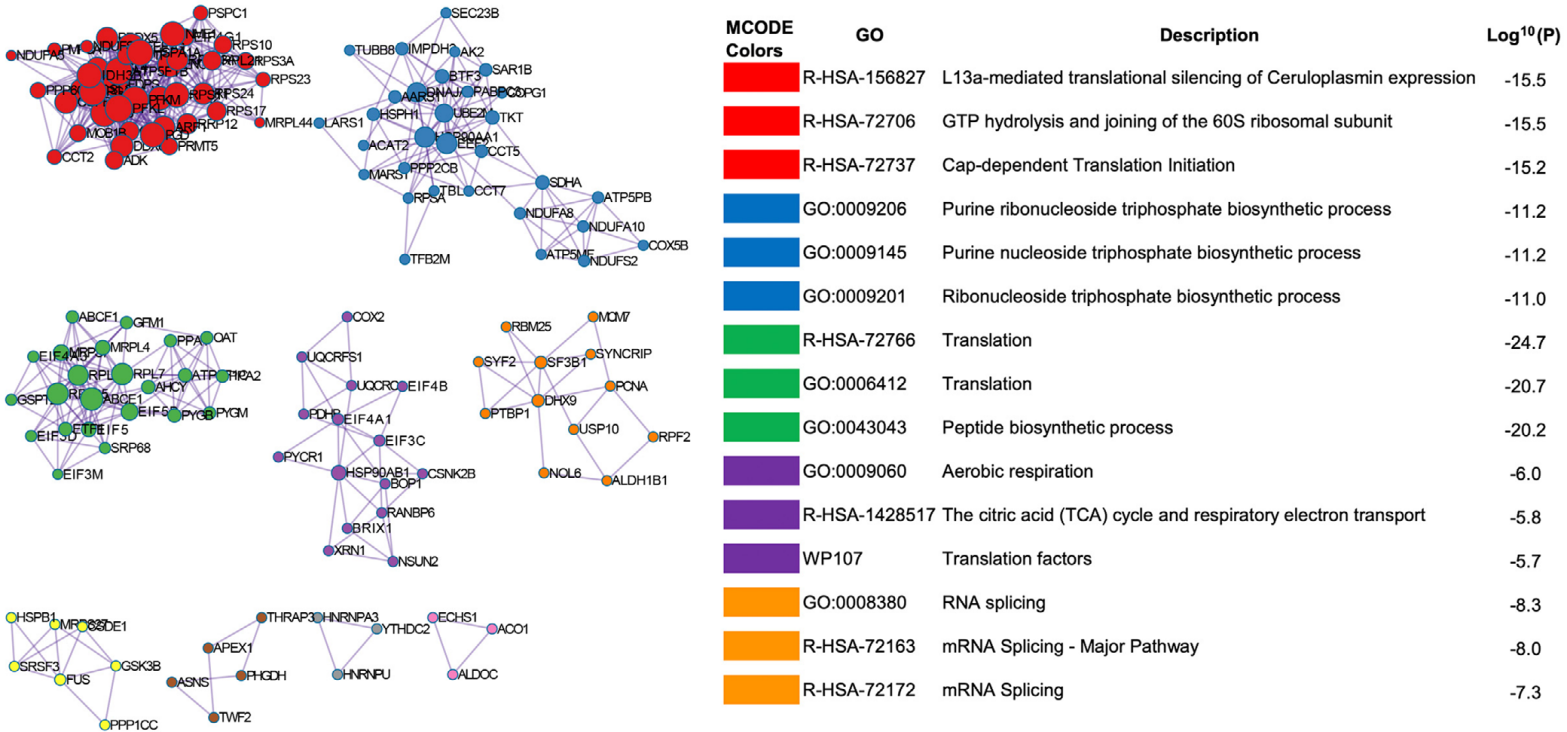
MCODE enrichment analysis performed using Metascape. Each colour represents a different MCODE protein–protein interaction (PPI) network.

We then performed MCODE enrichment analysis based on Protein–Protein Interaction (PPI) enrichment analysis for differentially expressed proteins using the default settings of the Metascape web portal. With PPI analysis, we observed network interactions of differentially expressed proteins between PC3‐P and PC3‐R cells in processes such as Ceruloplasmin expression in L13a‐mediated translational silencing [−Log10(*p*) = 15.5] (EIF4A2, EIF4G1, RPL21, RPL23A, etc.), purine ribonucleoside triphosphate biosynthetic processes [−Log10(*p*) = 11.2] (ATP5PB, IMPDH2, NDUFA8, etc.), translation [−Log10(*p*) = 24.7] (EIF5, ETF1, PPA1, RPL7, RPL38, SRP68, etc.), aerobic respiration [−Log10(*p*) = 6.0] (COX2, PDHB, UQCRC1, etc.), and RNA splicing [−Log10(*p*) = 8.3] (DHX9, PTBP1, SYNCRIP, etc.) (Figure 5). Interestingly, the RNA splicing MCODE component mostly includes up‐regulated proteins, while the other MCODE components are composed largely of down‐regulated proteins.

### 3.6 Comparison of Proteomic Data and Global Validated UALCAN Data

**FIGURE 6:**
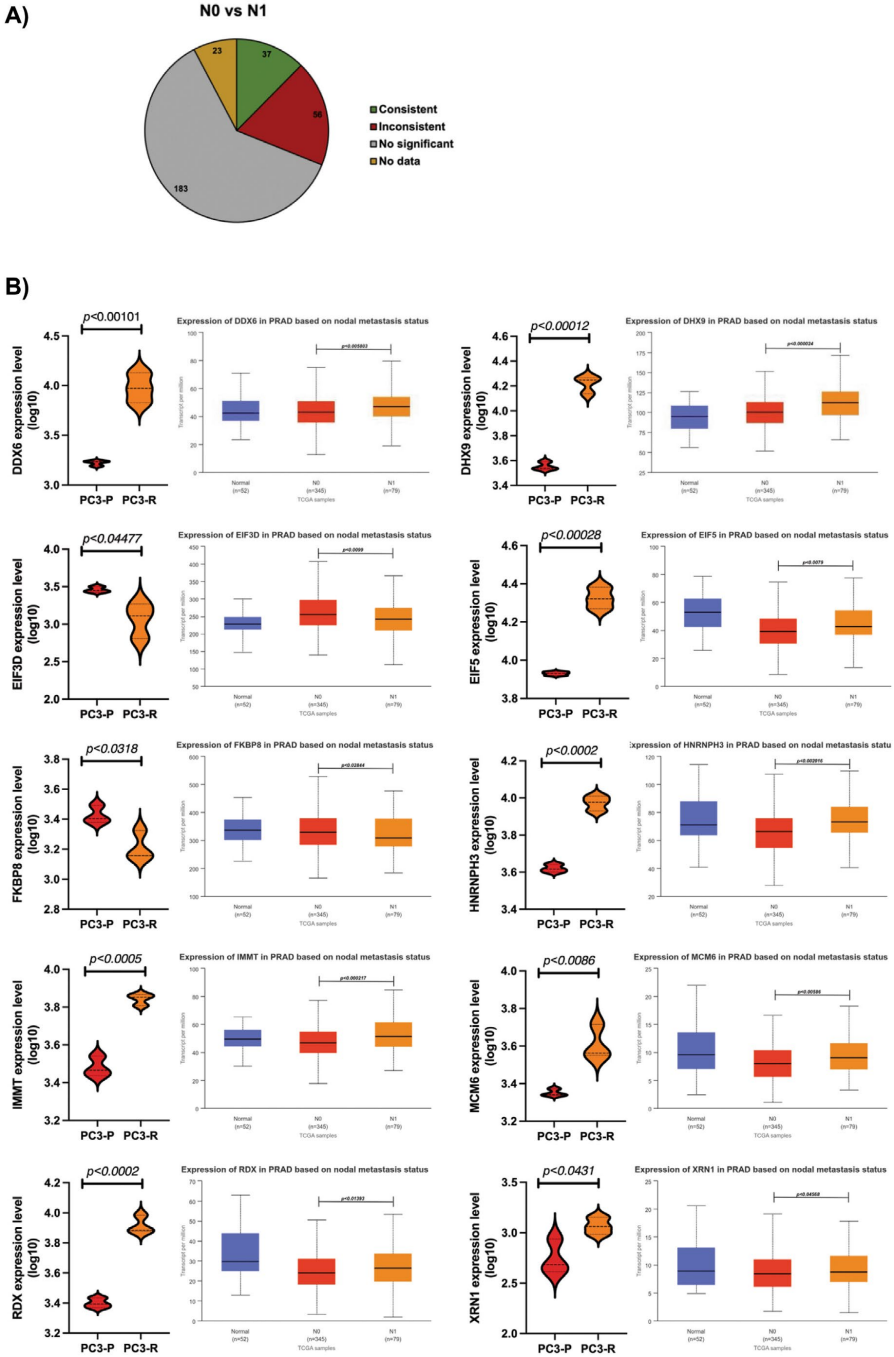
(A) Comparison of proteins with significantly changed expression according to nodal metastasis status in TCGA data. Thirty‐seven of 299 proteins were found consistent with N0 vs. N1 nodal status. While inconsistency was detected in 56 proteins, no statistical difference was observed in the TCGA N0 vs. N1 nodal status of 183 proteins. TCGA nodal metastasis status data is not available for 23 proteins. (B) Graphical comparison of the relative expressions of some of the proteins consistent with TCGA nodal metastasis status data.

**FIGURE 7:**
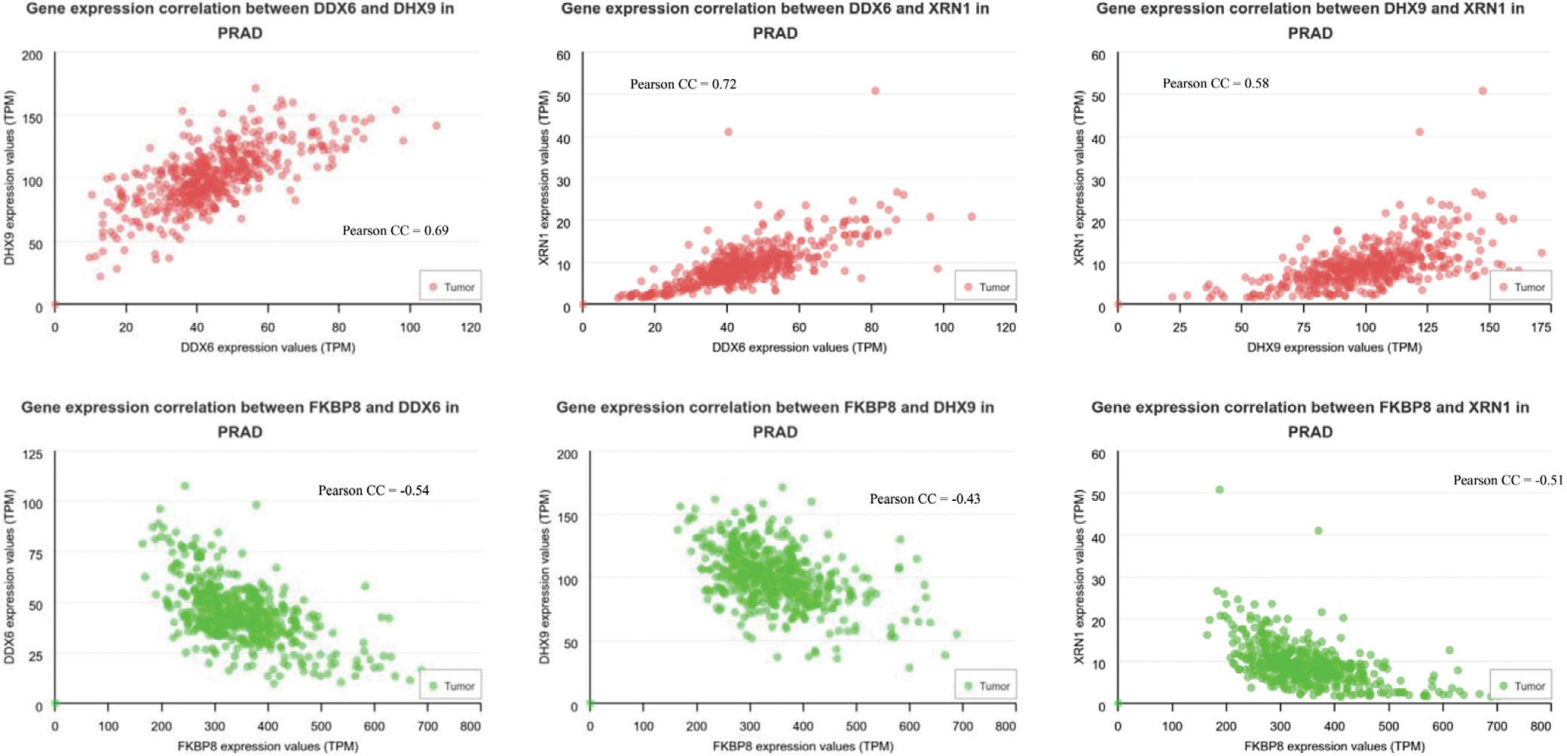
Correlations in gene expression between DHX9, DDX6, XRN1, and FKBP8. The analysis was conducted using prostate adenocarcinoma tumour samples from TCGA dataset via UALCAN data portal. Pearson CC refers to the Pearson correlation coefficient.

**FIGURE 8:**
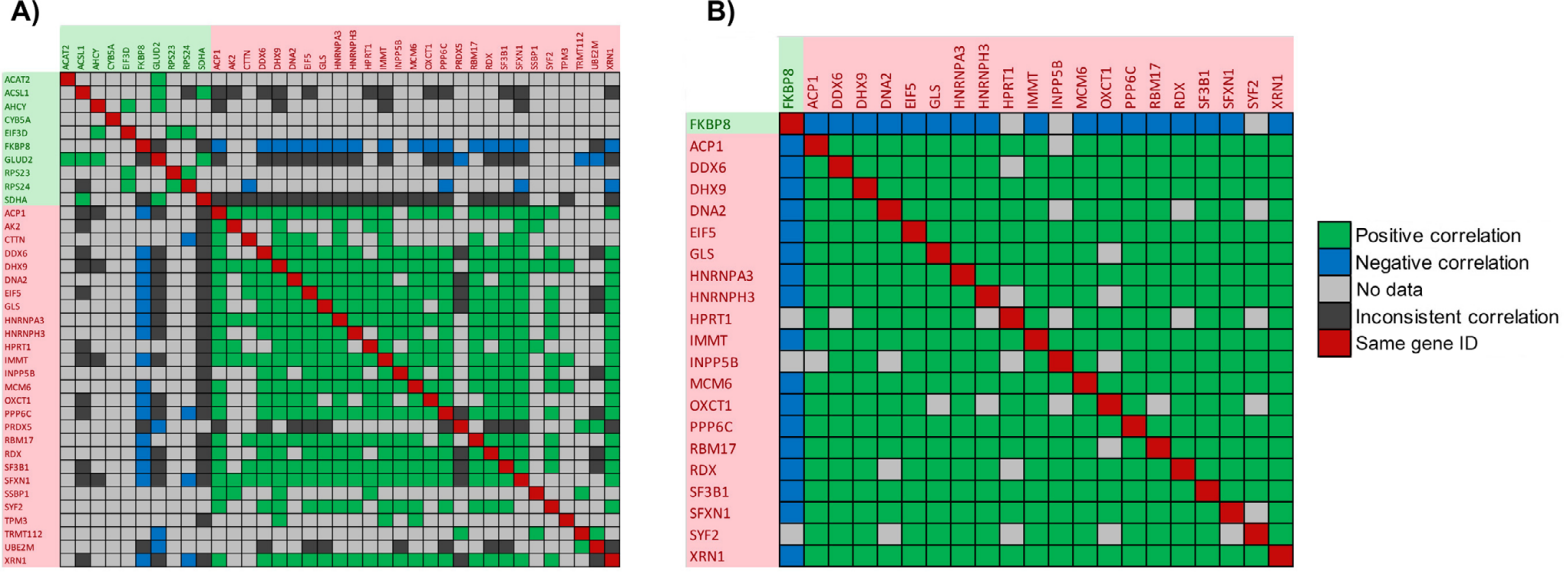
(A) Comparison of proteomic result correlations and TCGA correlations of 37 proteins consistent with TCGA nodal metastasis status. (B) Correlation graph resulting from filtering out proteins with more than half inconsistencies or no data after correlation comparison. Green coloured wells indicate that the relevant proteins are positively correlated in both the proteomic results and TCGA correlation data. Blue coloured wells indicate that the relevant proteins are negatively correlated in both the proteomic results and TCGA correlation data. Black coloured wells indicate that the proteomic and TCGA correlation data of the relevant proteins are inconsistent. Grey coloured wells indicate that TCGA correlation data is not available. Red coloured wells are the wells where the same IDs intersect.

Although studies showing a connection between acquired drug resistance and the metastatic properties of tumours exist [40, 41], no database exists concerning data sets of proteins whose expression is increased or decreased in cancer cells during drug resistance. About 90% of chemotherapy failures due to drug resistance occurring during cancer aggressiveness and metastasis [42]. We therefore explored the correlation between drug resistance and cancer metastasis by examining how our expression data aligns with TCGA nodal metastasis status. Our aim was to determine whether there is a relationship between the proteins that exhibit differential expression in bortezomib‐resistant cells and those implicated in the metastatic status of cancer cells. The UALCAN web portal allows researchers to access Level 3 RNA‐seq data from The Cancer Genome Atlas (TCGA) and perform gene expression and survival analysis on approximately 20,500 protein‐coding genes in 33 different tumour types [36]. We uploaded the genes significantly differentially expressed proteins between PC3‐P and PC3‐R cell lines to the UALCAN web portal link and analysed them with TCGA prostate adenocarcinoma datasets. We then examined the expression levels of each gene based on nodal metastasis status. According to the pathological N classification, N0 indicates no regional lymph node metastasis, N1 indicates metastasis in 1–3 lymph nodes, N2 indicates metastasis in 4–9 lymph nodes, and N3 indicates metastasis in 10 or more lymph nodes. No N2 and N3 status data were found in prostate adenocarcinoma nodal metastasis status. When the differentially expressed proteins between PC3‐P and PC3‐R cells were compared with TCGA N0 and N1 transcript differences based on nodal metastasis status, it was found that 37 proteins were concordant, 56 proteins were discordant, 183 proteins showed no statistical difference between N0 and N1 transcripts, and no data was showing a difference between N0 and N1 transcripts for 23 proteins (Figure 6A). The upregulated or downregulated proteins in the bortezomib‐resistant cells showing similar expression levels as compared to the metastatic process (i.e., from N0 to N1 stage) are represented in Figure 6B. For example, DHX9, EIF5, HNRNPH3, RDX, MCM6, IMMT, DDX6 and XRN1 proteins were also significantly upregulated during the course of the metastatic process. In contrast, FKBP8 and EIF3D proteins were similarly downregulated during the lymph nodal metastasis, which is consistent with our experimental data (Figure 6B). Consistent with these findings, the upregulated proteins DDX6, DHX9, and XRN1 exhibited significant positive correlations with each other in the TCGA tumour samples. In contrast, these upregulated proteins showed a negative correlation with the significantly downregulated FKBP8 protein, further supporting our experimental data (Figure 7). Subsequently, to specify and further validate these results, we compared the positive and negative correlations among the 37 proteins using UALCAN TCGA correlation datasets. In Figure 8A, the data with positive correlation consistent with both TCGA datasets and our proteomic data are shown as blue squares, data with negative correlation consistent with both TCGA datasets and our proteomic data are shown as green squares, the data inconsistent with both TCGA datasets and our proteomic data are shown as black squares. And the data with missing values in the TCGA datasets are shown as grey squares. It was determined that 21 proteins (DHX9, EIF5, HPRT1, INPP5B, MCM6, PPP6C, SYF2, etc.) whose expression was increased in PC3‐R cells based on label‐free nLC‐MS/MS analysis were consistent with both TCGA prostate adenocarcinoma N0 and N1 nodal metastasis status and correlation data (Figure 8B). Interestingly, FKBP8, whose expression was found to be decreased in PC3‐R cells in proteomic analysis, was the only protein consistently downregulated in both nodal metastasis status and correlation data. Among the proteins whose expression was increased in PC3‐R cells according to proteomic data, besides a few missing TCGA data, most showed positive correlation with each other and negative correlation with FKBP8.

## 4 Discussions

Chemotherapy remains an effective method for cancer treatment today. However, acquired resistance to chemotherapy is one of the major problems that negatively affect the success of treatment and reduce patients' survival. Therefore, the development of new methods to overcome this acquired resistance, better understanding of resistance mechanisms to chemotherapy, and discovery of new target molecules have become a necessity. Bortezomib, the first proteasome inhibitor approved by the FDA for the treatment of multiple myeloma, has achieved clinical success. Recent literature suggests that both bortezomib and novel proteasome inhibitors hold significant promise for the treatment of solid tumours [32]. However, acquired resistance following chemotherapy with bortezomib presents a major obstacle to its efficacy. In our study, we investigated the reasons for resistance to bortezomib at the molecular level in the PC3 prostate cancer cells as a model cell line.

In our previous studies, we obtained PC3‐Resistant cells with a 4.3‐fold higher resistance to bortezomib through dose escalation for six months [26]. In the current investigation, we observed that PC3‐Resistant cells subjected to sustained bortezomib exposure exhibited a remarkable 67‐fold increase in resistance to the drug. This underscores the progressive development of resistance to bortezomib within the PC3 prostate cancer cell line over time. Under our experimental conditions, we noted that resistant cells exhibit a slower rate of proliferation compared to parental cells, a phenomenon previously documented and linked to reduced levels of cyclin D1 [26]. This phenomenon is consistent with changes identified through bioinformatics research in translation initiation, purine triphosphate biosynthetic processes, mRNA stabilisation, and mitochondrial processes. The slower proliferation of cells suggests that they may be attempting to gain time for the required mechanisms for resistance by slowing down their metabolism. It is known that cells can enter the G0 phase of the cell cycle where there is no cell proliferation to develop drug resistance [43, 44]. Upon examining the changes in KEGG (Figure 4A) and REACTOME (Figure 4B) pathways, we observed that the majority of the changes involved the proteins with decreased expression, particularly those associated with metabolic and translational pathways in PC3‐resistant cells, we therefore hypothesised that PC3‐resistant cells may have entered the G0 phase to acquire resistance to bortezomib.

We also utilised metadata from UALCAN TCGA datasets to specify and verify our proteomic data. When comparing differentially expressed proteins with prostate adenocarcinoma TCGA data, we identified 37 proteins (ACAT2, EIF3D, FKBP8, DHX9, EIF5, HNRNPA3, PPP6C, SSMP1, UBE2M, etc.) that showed consistency between N0 versus N1 and PC3‐P versus PC3‐R data. To delineate the consistency of results, we compared the correlation metadata among these 37 proteins and found that 21 of them exhibited consistent correlations with each other. Interestingly, FKBP8 emerged as the only protein consistently downregulated in both nodal metastasis status and correlation data (Figure 8B). FKBP8 is a newly identified protein involved in positively regulating the autophagic pathway [45]. This suggests that FKBP8 may play a critical role in both resistance mechanisms and metastatic conditions, and may be a potential target for combination studies involving proteasome inhibitors. Furthermore, we identified XRN1 (a hexoribonuclease) and DDX6 (a helicase) as proteins whose expression was increased in PC3‐R cells and was consistent with UALCAN TCGA data. Both XRN1 and DDX6 are molecules involved in mRNA decay and suppression of autophagy [46]. The increased expression of XRN1 and DDX6 in PC3‐R cell line suggests their role in acquired resistance to bortezomib by enhancing mRNA decay and suppressing autophagy. This implies that targeting XRN1 and DDX6 could potentially overcome resistance developed against bortezomib. Moreover, DHX9, a dsRNA helicase enriched in intron RNA damage, supports cell survival and stimulates dsRNA‐associated immune response and translational shutdown [47]. In response to increased mRNA decay mediated by XRN1 and DDX6, DHX9 emerges as a significant target molecule in acquired resistance to bortezomib in PC3 prostate cancer cells [47]. In conclusion, this study identifies FKBP8 as consistently downregulated and DDX6, XRN1, and DHX9 as consistently upregulated molecules in bortezomib‐resistant PC3‐R cells, playing crucial roles in mRNA decay, translational suppression, and autophagy suppression. Targeting DDX6, XRN1, and DHX9 could serve as potential therapeutic strategies to overcome resistance developed during bortezomib treatment in clinical settings.

## Author Contributions

**Semih Seker:** conceptualization (equal), data curation (equal), formal analysis (equal), investigation (equal), writing – original draft (equal), writing – review and editing (equal). **Betul Sahin:** data curation (equal), investigation (equal). **Azmi Yerlikaya:** conceptualization (lead), data curation (equal), formal analysis (equal), funding acquisition (equal), investigation (equal), project administration (lead), writing – original draft (equal), writing – review and editing (equal).

## Ethics Statement

The study did not involve experimental animals, patient tissues, or primary cell lines; therefore, it does not require ethical approval from local committees.

## Conflicts of Interest

The authors decare no conflicts of interest.

## Supporting information


Table S1.

## Data Availability

Data available on request from the authors.

## References

[1] F. Wang , L. A. Canadeo , and J. M. Huibregtse , “Ubiquitination of Newly Synthesized Proteins at the Ribosome,” Biochimie 114 (2015): 127–133, 10.1016/j.biochi.2015.02.006. 25701549 PMC4458410

[2] C. Pohl and I. Dikic , “Cellular Quality Control by the Ubiquitin‐Proteasome System and Autophagy,” Science 366 (2019): 818–822. 31727826 10.1126/science.aax3769

[3] J. D. Etlinger and A. L. Goldberg , “A Soluble ATP‐Dependent Proteolytic System Responsible for the Degradation of Abnormal Proteins in Reticulocytes,” Proceedings of the National Academy of Sciences of the United States of America 74 (1977): 54–58. 264694 10.1073/pnas.74.1.54 PMC393195

[4] H. He , X. M. Qi , J. Grossmann , and C. W. Distelhorst , “C‐Fos Degradation by the Proteasome. An Early, Bcl‐2‐Regulated Step in Apoptosis,” Journal of Biological Chemistry 273, no. 39 (1998): 25015–25019. 9737957 10.1074/jbc.273.39.25015

[5] W. Hilt and D. H. Wolf , “Proteasomes: Destruction as a Programme,” Trends in Biochemical Sciences 21, no. 3 (1996): 96–102. 8882582

[6] J. Adams , “The Proteasome: A Suitable Antineoplastic Target,” Nature Reviews Cancer 4, no. 5 (2004): 349–360. 15122206 10.1038/nrc1361

[7] S. Lü and J. Wang , “The Resistance Mechanisms of Proteasome Inhibitor Bortezomib,” Biomarker Research 1 (2013): 1–9. 24252210 10.1186/2050-7771-1-13 PMC4177604

[8] A. Yerlikaya and M. Yontem , “The Significance of Ubiquitin Proteasome Pathway in Cancer Development,” Recent Patents on Anti‐Cancer Drug Discovery 8 (2013): 298–309. 23061719 10.2174/1574891x113089990033

[9] A. Hershko and A. Ciechanover , “The Ubiquitin System,” Annual Review of Biochemistry 67 (1998): 425–479. 10.1146/annurev.biochem.67.1.425 9759494

[10] E. G. Mimnaugh , H. Y. Chen , J. R. Davie , J. E. Cells , and L. Neckers , “Rapid Deubiquitination of Nucleosomal Histones in Human Tumor Cells Caused by Proteasome Inhibitors and Stress Response Inducers: Effects on Replication, Transcription, Translation, and the Cellular Stress Response,” Biochemistry 36 (1997): 14418–14429. 9398160 10.1021/bi970998j

[11] P. E. Stoebner , T. Lavabre‐Bertrand , L. Henry , et al., “High Plasma Proteasome Levels Are Detected in Patients With Metastatic Malignant Melanoma,” British Journal of Dermatology 152 (2005): 948–953. 15888151 10.1111/j.1365-2133.2005.06487.x

[12] L. Chen and K. Madura , “Increased Proteasome Activity, Ubiquitin‐Conjugating Enzymes, and eEF1A Translation Factor Detected in Breast Cancer Tissue,” Cancer Research 65 (2005): 5599–5606. 15994932 10.1158/0008-5472.CAN-05-0201

[13] A. Arlt , I. Bauer , C. Schafmayer , et al., “Increased Proteasome Subunit Protein Expression and Proteasome Activity in Colon Cancer Relate to an Enhanced Activation of Nuclear Factor E2‐Related Factor 2 (Nrf2),” Oncogene 28, no. 45 (2009): 3983–3996. 19734940 10.1038/onc.2009.264

[14] R. C. Kane , P. F. Bross , A. T. Farrell , and R. Pazdur , “Velcade®: U.S. FDA Approval for the Treatment of Multiple Myeloma Progressing on Prior Therapy,” Oncologist 8, no. 6 (2003): 508–513. 14657528 10.1634/theoncologist.8-6-508

[15] R. C. Kane , R. Dagher , A. Farrell , et al., “Bortezomib for the Treatment of Mantle Cell Lymphoma,” Clinical Cancer Research 13 (2007): 5291–5294. 17875757 10.1158/1078-0432.CCR-07-0871

[16] C. R. Berkers , M. Verdoes , E. Lichtman , et al., “Activity Probe for In Vivo Profiling of the Specificity of Proteasome Inhibitor Bortezomib,” Nature Methods 2 (2005): 357–362. 15846363 10.1038/nmeth759

[17] S. Narayanan , C. Y. Cai , Y. G. Assaraf , et al., “Targeting the Ubiquitin‐Proteasome Pathway to Overcome Anti‐Cancer Drug Resistance,” Drug Resistance Updates 48 (2020): 100663. 31785545 10.1016/j.drup.2019.100663

[18] G. Kozalak , İ. Bütün , E. Toyran , and A. Koşar , “Review on Bortezomib Resistance in Multiple Myeloma and Potential Role of Emerging Technologies,” Pharmaceuticals 16 (2023): 111. 36678608 10.3390/ph16010111 PMC9864669

[19] A. Yerlikaya , S. Altıkat , R. Irmak , et al., “Effect of Bortezomib in Combination With Cisplatin and 5‐Fluorouracil on 4T1 Breast Cancer Cells,” Molecular Medicine Reports 8 (2013): 277–281. 23660746 10.3892/mmr.2013.1466

[20] J. Zhao , C. Wang , Y. Song , Y. Liu , and B. Fang , “Treatment of Refractory/Relapsed Adult Acute Lymphoblastic Leukemia With Bortezomib‐Based Chemotherapy,” International Journal of General Medicine 8 (2015): 211–214. 26109875 10.2147/IJGM.S59537 PMC4472074

[21] S. Lü , J. Yang , X. Song , et al., “Point Mutation of the Proteasome Beta5 Subunit Gene Is an Important Mechanism of Bortezomib Resistance in Bortezomib‐Selected Variants of Jurkat T Cell Lymphoblastic Lymphoma/Leukemia Line,” Journal of Pharmacology and Experimental Therapeutics 326 (2008): 423–431. 18502982 10.1124/jpet.108.138131

[22] R. Oerlemans , N. E. Franke , Y. G. Assaraf , et al., “Molecular Basis of Bortezomib Resistance: Proteasome Subunit β5 (PSMB5) Gene Mutation and Overexpression of PSMB5 Protein,” Blood 112 (2008): 2489–2499. 18565852 10.1182/blood-2007-08-104950

[23] E. Suzuki , S. Demo , E. Deu , et al., “Molecular Mechanisms of Bortezomib Resistant Adenocarcinoma Cells,” PLoS One 6 (2011): e27996. 22216088 10.1371/journal.pone.0027996 PMC3245226

[24] N. E. Franke , D. Niewerth , Y. G. Assaraf , et al., “Impaired Bortezomib Binding to Mutant β5 Subunit of the Proteasome Is the Underlying Basis for Bortezomib Resistance in Leukemia Cells,” Leukemia 26 (2012): 757–768. 21941364 10.1038/leu.2011.256

[25] Y. X. Wu , J. H. Yang , and H. Saitsu , “Bortezomib‐Resistance Is Associated With Increased Levels of Proteasome Subunits and Apoptosis‐Avoidance,” Oncotarget 7 (2016): 77622–77634. 27769058 10.18632/oncotarget.12731 PMC5363609

[26] A. Yerlikaya and E. Okur , “An Investigation of the Mechanisms Underlying the Proteasome Inhibitor Bortezomib Resistance in PC3 Prostate Cancer Cell Line,” Cytotechnology 72 (2020): 121–130. 31863311 10.1007/s10616-019-00362-x PMC7002631

[27] K. Zafeiropoulou , G. Kalampounias , S. Alexis , D. Anastasopoulos , A. Symeonidis , and P. Katsoris , “Autophagy and Oxidative Stress Modulation Mediate Bortezomib Resistance in Prostate Cancer,” PLoS One 19 (2024): e0289904. 38412186 10.1371/journal.pone.0289904 PMC10898778

[28] M. M. Bradford , “A Rapid and Sensitive Method for the Quantitation of Microgram Quantities of Protein Utilizing the Principle of Protein‐Dye Binding,” Analytical Biochemistry 72 (1976): 248–254. 942051 10.1016/0003-2697(76)90527-3

[29] I. Kiris , K. Skalicka‐Wozniak , M. K. Basar , B. Sahin , B. Gurel , and A. T. Baykal , “Molecular Effects of Pteryxin and Scopoletin in the 5xFAD Alzheimer's Disease Mouse Model,” Current Medicinal Chemistry 29 (2021): 2937–2950. 10.2174/0929867328666210827152914 34455957

[30] E. Akgun , M. B. Tuzuner , B. Sahin , et al., “Proteins Associated With Neutrophil Degranulation Are Upregulated in Nasopharyngeal Swabs From SARS‐CoV‐2 Patients,” PLoS One 15 (2020): e0240012. 33079950 10.1371/journal.pone.0240012 PMC7575075

[31] S. Zeren , S. Seker , G. A. Akgün , E. Okur , and A. Yerlikaya , “Label‐Free nLC‐MS/MS Proteomic Analysis Reveals Significant Differences in the Proteome Between Colorectal Cancer Tissues and Normal Colon Mucosa,” Medical Oncology 40 (2023): 1–12. 10.1007/s12032-023-02173-9 37707637

[32] E. Okur and A. Yerlikaya , “A Novel and Effective Inhibitor Combination Involving Bortezomib and OTSSP167 for Breast Cancer Cells in Light of Label‐Free Proteomic Analysis,” Cell Biology and Toxicology 35 (2019): 33–47. 29948483 10.1007/s10565-018-9435-z

[33] B. T. Sherman , M. Hao , J. Qiu , et al., “DAVID: A Web Server for Functional Enrichment Analysis and Functional Annotation of Gene Lists (2021 Update),” Nucleic Acids Research 50 (2022): W216–W221. 35325185 10.1093/nar/gkac194 PMC9252805

[34] D. Tang , M. Chen , X. Huang , et al., “SRplot: A Free Online Platform for Data Visualization and Graphing,” PLoS One 18 (2023): e0294236. 37943830 10.1371/journal.pone.0294236 PMC10635526

[35] Y. Zhou , B. Zhou , L. Pache , et al., “Metascape Provides a Biologist‐Oriented Resource for the Analysis of Systems‐Level Datasets,” Nature Communications 10 (2019): 1523. 10.1038/s41467-019-09234-6 PMC6447622 30944313

[36] D. S. Chandrashekar , S. K. Karthikeyan , P. K. Korla , et al., “UALCAN: An Update to the Integrated Cancer Data Analysis Platform,” Neoplasia 25 (2022): 18–27. 35078134 10.1016/j.neo.2022.01.001 PMC8788199

[37] E. A. Zaal , H. J. de Grooth , I. Oudaert , et al., “Targeting Coenzyme Q10 Synthesis Overcomes Bortezomib Resistance in Multiple Myeloma,” Molecular Omics 18 (2022): 19–30. 34879122 10.1039/d1mo00106j

[38] M. Kanehisa , S. Goto , Y. Sato , M. Furumichi , and M. Tanabe , “KEGG for Integration and Interpretation of Large‐Scale Molecular Data Sets,” Nucleic Acids Research 40 (2012): D109–D114. 22080510 10.1093/nar/gkr988 PMC3245020

[39] M. Gillespie , B. Jassal , R. Stephan , et al., “The Reactome Pathway Knowledgebase 2022,” Nucleic Acids Research 50 (2022): D687–D692. 34788843 10.1093/nar/gkab1028 PMC8689983

[40] R. S. Kerbel , H. Kobayashi , and C. H. Graham , “Intrinsic or Acquired Drug Resistance and Metastasis: Are They Linked Phenotypes?,” Journal of Cellular Biochemistry 56 (1994): 37–47. 7806590 10.1002/jcb.240560108

[41] N. Ebrahimi , M. S. Manavi , F. Faghihkhorasani , et al., “Harnessing Function of EMT in Cancer Drug Resistance: A Metastasis Regulator Determines Chemotherapy Response,” Cancer Metastasis Reviews 43 (2024): 457–479. 38227149 10.1007/s10555-023-10162-7

[42] B. Mansoori , A. Mohammadi , S. Davudian , S. Shirjang , and B. Baradaran , “The Different Mechanisms of Cancer Drug Resistance: A Brief Review,” Advanced Pharmaceutical Bulletin 7 (2017): 339–348. 29071215 10.15171/apb.2017.041 PMC5651054

[43] A. J. Wiecek , S. J. Cutty , D. Kornai , et al., “Genomic Hallmarks and Therapeutic Implications of G0 Cell Cycle Arrest in Cancer,” Genome Biology 24 (2023): 1–35. 37221612 10.1186/s13059-023-02963-4 PMC10204193

[44] E. Lindell , L. Zhong , and X. Zhang , “Quiescent Cancer Cells‐A Potential Therapeutic Target to Overcome Tumor Resistance and Relapse,” International Journal of Molecular Sciences 24 (2023): 3762. 36835173 10.3390/ijms24043762 PMC9959385

[45] M. O. Aguilera , E. Robledo , M. Melani , P. Wappner , and M. I. Colombo , “FKBP8 Is a Novel Molecule That Participates in the Regulation of the Autophagic Pathway,” Biochimica et Biophysica Acta (BBA)—Molecular Cell Research 1869 (2022): 119212. 35090967 10.1016/j.bbamcr.2022.119212

[46] G. Hu , T. McQuiston , A. Bernard , et al., “A Conserved Mechanism of TOR‐Dependent RCK‐Mediated mRNA Degradation Regulates Autophagy,” Nature Cell Biology 17 (2015): 930–942. 26098573 10.1038/ncb3189 PMC4528364

[47] Y. Zhou , A. Panhale , M. Shvedunova , et al., “RNA Damage Compartmentalization by DHX9 Stress Granules,” Cell 187 (2024): 1701–1718. 38503283 10.1016/j.cell.2024.02.028

